# Mitigating Severe Ribociclib‐Hepatotoxicity With Corticosteroids

**DOI:** 10.1155/crom/4222126

**Published:** 2026-04-24

**Authors:** Julie Williamson, Maria Pletneva, Rebecca G. Kim, Roma Bhatia

**Affiliations:** ^1^ Division of Medical Oncology, Huntsman Cancer Institute, University of Utah, Salt Lake City, Utah, USA, utah.edu; ^2^ Department of Pathology, University of Utah, Salt Lake City, Utah, USA, utah.edu; ^3^ Division of Gastroenterology and Hepatology, Department of Internal Medicine, University of Utah, Salt Lake City, Utah, USA, utah.edu

## Abstract

Ribociclib adds progression‐free survival and overall survival benefit in combination with endocrine therapy for first‐line treatment of advanced HR+ HER2− breast cancer; however, it comes with a risk of hepatotoxicity. There is guidance for monitoring liver function and holding the medication if hepatotoxicity arises. However, there is minimal guidance for management if withholding the medication alone is insufficient. We present a case of a woman with de novo metastatic HR+ HER2− breast cancer who developed grade 2 AST and ALT elevation after two cycles of ribociclib and anastrozole, which progressed over several weeks to Grade 4 hepatotoxicity despite holding the medication. Extensive evaluation revealed drug‐induced liver injury from ribociclib as the cause. Ultimately, a long course of corticosteroids was initiated with remarkable response and resolution of transaminitis. An empiric trial of corticosteroids should be considered for patients with severe ongoing hepatotoxicity from CDK4/6 inhibitors despite cessation of the drug.

## 1. Introduction

Cyclin‐dependent kinase 4 and 6 (CDK4/6) inhibitors have emerged as frontline therapy in combination with endocrine therapy for the treatment of metastatic hormone receptor positive (HR+) human epidermal growth factor negative (HER2−) breast cancer [1–3]. Additionally, at least two of them have demonstrated a durable reduction in recurrence risk; therefore expanding their indication to the adjuvant setting [4, 5].

Transaminitis has been noted in all trials evaluating CDK4/6 inhibitors compared with controls and thus has been well characterized in the literature. A meta‐analysis noted a pooled relative risk of any grade AST elevation of 2.18, with 13.1% for those receiving CDK4/6 inhibitors versus 5.4% of controls. Similarly, there was a relative risk of 2.7 for Grade 3–4 AST elevation (2.9% vs. 0.9%) and a relative risk of 4.43 for Grade 3‐4 ALT elevation (4.1% vs. 0.8%) [6]. Ribociclib is the only CDK4/6 inhibitor that has extended overall survival in the first‐line setting and is the only medication with a Category 1 recommendation per NCCN guidelines.

However, of all the FDA approved CDK4/6 inhibitors in metastatic breast cancer, ribociclib comes with the greatest risk for hepatotoxicity. Approximately 11% of patients on ribociclib experience Grade 3 or 4 liver toxicity, with 8%–9% of patients discontinuing the drug permanently as a result [3, 5]. A pharmacovigilance study analyzing the FDA Adverse Event Reporting System (FAERS) database identified a significant association between ribociclib and drug induced liver injury (DILI), with a reporting odds ratio of 2.60, which was the highest risk compared with other CDK4/6 inhibitors. The median onset of DILI was 42 days [7]. Monitoring liver function tests during treatment is recommended and has clear guidance. Additionally, aside from general factors that increase risk of hepatotoxicity (drugs), there are no clear predictors or known associations of patients on ribociclib who end up developing hepatotoxicity, making it difficult to understand how to prevent the complication in the first place. However, guidance on treatment of DILI is limited and aside from holding or reducing the dose of the drug as guided by package inserts, there are no formal recommendations on how to resolve this complication, particularly in patients who do experience Grade 3–4 hepatotoxicity.

## 2. Case Presentation

A premenopausal woman in her 50s was diagnosed with de novo, metastatic, strongly ER/PR+ HER2− invasive ductal carcinoma with bone metastasis confirmed by biopsy. She was started on first line combination aromatase inhibitor and CDK4/6 inhibitor with anastrozole and ribociclib, as well as ovarian suppression with goserelin. She developed Grade 2 transaminitis prior to Cycle 3, so ribociclib was held. Despite holding the medication, her liver injury progressed to Grade 4 AST and ALT elevation over several weeks.

**Figure 1:**
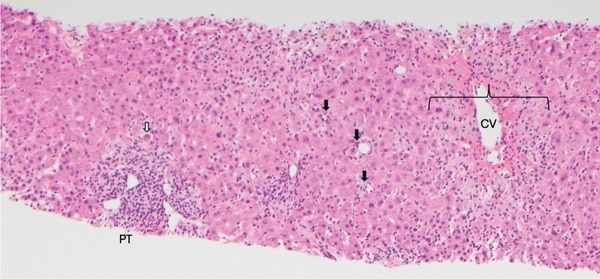
Liver biopsy, H&E 100X. The most prominent histologic findings included marked lobular disarray with scattered acidophil bodies (white arrow) and lymphohistiocytic aggregates (black arrows), moderate periportal predominantly lymphocytic inflammation without significant interface activity, and centrizonal necrosis with debris‐laden histiocytes (bracket), consistent with active hepatitis. In the setting of the patient′s clinical picture and ribociclib treatment, histologic findings are most likely due to drug‐induced liver injury. CV: central vein. PT: portal tract.

An extensive evaluation to work up her hepatocellular injury was done during her hospitalization. MRI abdomen was unremarkable and without any metastatic disease in the liver. Infectious workup was negative including hepatitis A, hepatitis B, hepatitis C, hepatitis E, HSV, EBV, VZV, and CMV. Autoimmune hepatitis was evaluated with ANA, F‐actin antibody, mitochondrial M2 antibody, and immunoglobulins, which were all within normal limits. Alpha‐1‐antitrypsin and ceruloplasmin were also within normal limits. Drug panel was negative and the patient denied any recent alcohol or acetaminophen use. She had no other antecedent viral illnesses. Ultimately, a liver biopsy was done, which showed periportal and lobular inflammation with hepatocyte necrosis consistent with active hepatitis, and drug‐induced liver injury was favored (Figure 1).

Her transaminitis peaked at AST 1058, ALT 2379 1 month since her last dose of ribociclib and after 3 months total of ribociclib and initially started to downtrend; however, levels began to rise again 1 week later. In consultation with hepatology and based on prior case reports of severe transaminitis associated with CDK4/6 inhibitors, the decision was made to trial corticosteroids with prednisone 60 mg/day [8].

**Figure 2:**
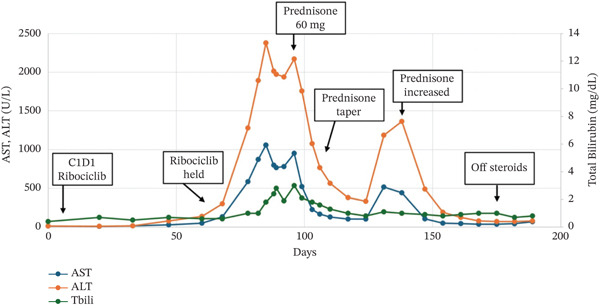
Patient′s liver enzyme trend and clinical course.

Her liver enzymes began improving within 2 days. Prednisone was slowly tapered over several weeks with continued response. When the dose was reduced to 20 mg/day, her transaminitis began to worsen again. Her anastrozole, which had been held since her initial Grade 4 AST/ALT elevation, was restarted 2 weeks prior to this. Anastrozole was held again and prednisone was increased back to 40 mg/day with response within 1–2 weeks and was slowly tapered over the following 1.5 months as transaminases normalized. Synthetic liver function remained intact, with normal PT/INR, platelets, bilirubin and albumin. Clinical course and liver enzyme trend outlined in Figure 2 and Table 1. Six months after discontinuation of ribociclib, palbociclib and letrozole were initiated without change in liver enzymes, and she has been tolerating well with stable disease.

**Table 1:** Patient′s liver enzyme trend and clinical course.

Day	AST (U/L)	ALT (U/L)	Total bilirubin (mg/dL)	Medication changes
0.00	12	13	0.4	C1 ribociclib
20.00	8	9	0.7	
33.00	14	16	0.5	C2 ribociclib
47.00	28	80	0.7	
60.00	49	138	0.6	C3 ribociclib held
68.00	132	300	0.6	
78.00	586	1279	1	Anastrozole discontinued
82.00	874	1893	1	
85.00	1058	2379	1.8	
88.00	800	2013	2.4	
89.00	767	1974	2.8	
92.00	782	1940	1.9	
96.00	952	2171	3	Prednisone 60 mg daily
99.00	523	1757	2.1	
103.00	226	1078	1.8	Prednisone taper, decrease by 10 mg per week
106.00	168	767	1.6	
110.00	130	564	1.3	
117.00	103	380	1	Anastrozole restarted
124.00	102	332	0.8	Prednisone decreased to 20 mg daily
131.00	517	1187	1.1	Prednisone increased to 40 mg, anastrozole held
138.00	442	1366	1	
147.00	105	489	0.9	
154.00	49	192	0.8	
161.00	44	124	0.9	
168.00	37	80	1	
175.00	37	71	1	Off steroids
182.00	43	71	0.7	
189.00	68	79	0.8	
203.00	39	55	0.9	

## 3. Discussion

Hepatotoxicity is a common adverse effect with use of ribociclib and warrants close monitoring particularly in the first several months of drug initiation. AST and ALT elevation often improve with withholding the drug, although this can take time, often 3–5 months to normalize [9]. In the event transaminases fail to normalize or continue to worsen despite withholding the medication, as it did in this clinical case, treatment is unclear. One retrospective study in France identified 22 patients with grade 3–4 hepatitis induced by CDK4/6 inhibitors, 9 of which were treated with corticosteroids. Though time to resolution was slower for patients treated with corticosteroids, these patients had a trend toward greater degree of liver enzyme elevation, had more liver biopsies, and minimal improvement after discontinuation of the CDK4/6 inhibitor. In all patients, liver enzymes significantly reduced within 7 days of corticosteroids [8].

The clinical case described here demonstrates continued significant progression of AST and ALT elevation for 1 month since drug discontinuation, with rapid improvement with corticosteroids. Anastrozole was held during initial Grade 4 AST/ALT elevation and was restarted 2 weeks before subsequent rise in AST/ALT, so this was considered as a possible etiology. However, the risk of DILI from anastrozole is rare (2%–4%) and generally mild, rarely requiring intervention or dose modification [10]. It is much more likely due to ribociclib‐induced liver injury, and the rise in AST/ALT occurred during corticosteroid taper and quickly resolved with an increase in corticosteroid dose and a slower taper.

The role of corticosteroids for generalized drug‐induced liver injury is unclear, with mixed success in the absence of autoimmune hepatitis [11]. The American Association for the Study of Liver Diseases (AASLD) practice guidelines recommend an initial prednisone dose of 40–60 mg for select cases of idiosyncratic drug‐induced liver injury, although guidance is unclear outside of use in tyrosine kinase inhibitors or immunotherapy‐induced liver injury [12]. However, the success and rapid effect of corticosteroids highlight that these agents should be considered in severe transaminitis from CDK4/6 inhibitors, of which the highest frequency culprit is ribociclib.

## Funding

No funding was received for this manuscript.

## Conflicts of Interest

The authors declare no conflicts of interest.

## Data Availability

The data that support the findings of this study are available on request from the corresponding author. The data are not publicly available due to privacy or ethical restrictions.
